# Mr. Dean's Case of Uterine Cancer

**Published:** 1802-11-01

**Authors:** W. Nisbet


### To Dr. BATTY.

DEAR SIR,

THE two following cafes, noticed in the laft Journal, I fend you as drawn up by the attending Surgeons.

I am, &c.

W. NISBET.

---

Cafe I. *Communicated by* Mr. BEVERIDGE, *Member of the Royal College of Surgeons, London.*

Mrs. U. of Ludgate Hill, fome months after parturition, was attacked by a fwelling of the external parts, hard, apparently fchirrous, and foon becoming exquifitely painful. This affection continued for no lefs a period than fix years under a variety of medical treatment, and then difappeared, but was immediately fucceeded by fymptoms of its being transferent to the uterus. Thefe fymptoms were violent pains of back and loins, fenfe of conftant weight and preffure, uneafinefs in going to ftool, inability to admit coition, a conftant acrid ferous difcharge, &c. Thefe fymptoms progreffively advanced, fo as to render the life of the patient completely miferable and ufelefs to her family. During the courfe of her malady, a number of different phyficians were confulted, whofe names it would be needlefs to mention without permiffion. Their opinions uniformly concurred in the nature of the difeafe, and in the flight profpect afforded of relief. In this ftate of matters, hearing accidentally of Dr. Nifbet, I advifed his being called in. On being made acquainted with the hiftory of the cafe, and after examining the actual ftate of the difeafe, which fhowed the uterus enlarged, hard, and painful to the touch, with the vagina conveying a hard glaffy feel, Dr. Nifbet's opinion concurred with the other gentlemen in the nature of the difeafe, giving however fome hopes of relief, and enjoining a mode of treatment different from what had been attempted in regard to its principle. As the patient, in the progrefs of her malady, had been unhappily expofed to venereal infection, with a view to this circumftance a pill was prefcribed, compofed of three parts of fteel to one of mercury, the minerals being prepared by depriving them as much as poffible of their oxygen, and exhibited in their leaft active ftate; along with this, the ufe of narcotics was enjoined, and the cicuta preferred for this purpofe. It was prepared in the fame manner, by reducing it to a carbonated ftate, and giving it in the form of draught, to to the extent of two drachms every day, with a few drops of effential oil to make it eafy on the ftomach. With this treatment the carbonic acid was largely thrown in, and a dilute vegetable diet recommended. To palliate the fymptoms of pain of back and loins in the firft inftance, a ftrong camphorated ointment, in the proportion of two and even three drachms to the ounce of axunge, was defired to be rubbed in night and morning, in the fame manner as practifed with the mercurial ointment. The good effects of this practice were foon apparent; the pain of back and loins, after the firft eight days, gradually decreafed; the uterine pain and preffure were removed; the patient was able to move about and attend her family concerns; lately, fhe even walked from her own houfe to Fitzroy-Square. Two of the gentlemen who formerly attended her, accidentally meeting her, expreffed their ftrong furprize at the change of the appearance; there is, therefore, every well grounded hope of her permanent cure.

*Oct.* 10, *Newgate-Street.*

Cafe II. *Communicated by* Mr. Dean, *Fellow of the Royal College of Surgeons, London.*

Mrs. H's complaint arofe from a mifcarriage, which took place a few months after her marriage, ten years ago. From this period, fhe was troubled with complaints in the abdomen, which induced her to confult Dr. P. He confidered thefe fymptoms as produced by a difeafed uterus, and advifed her to take the opinion of fome experienced phyfician in Midwifery. This, however, fhe neglected for a year and a half, when the fymptoms having much increafed, fhe came to London for a confultation on her complaint. On feeing her, I advifed her calling in Dr. G.; he found on examining the uterus, that it was enlarged, very hard, and that the fundus was thruft down into the lower part of the pelvis, fo as to prefs on the rectum, and it was fo expanded, that it could not be moved by the finger from its fituation; the complaint was attended with a moft excruciating pain on going to ftool, and moft of the other fymptoms defcribed in the former cafe. Dr. G. then propofed confulting with Dr. Nifbet, and that he fhould fee her. Having afcertained the nature of the difeafe, Dr. Nifbet propofed the fame plan of treatment as in the preceding cafe; and the fame advantages attended its application. The uterus has diminifhed in fize, the fymptoms in the abdomen and furrounding parts are much gone, and there is a well-grounded hope that the difeafe is faft removing.

*Oxford Street, October* 10, 1802.

